# Brassinosteroid-mediated stress tolerance in Arabidopsis shows interactions with abscisic acid, ethylene and salicylic acid pathways

**DOI:** 10.1186/1471-2229-10-151

**Published:** 2010-07-19

**Authors:** Uday K Divi, Tawhidur Rahman, Priti Krishna

**Affiliations:** 1 Department of Biology, University of Western Ontario, London, ON, N6A 5B7, Canada

## Abstract

**Background:**

Brassinosteroids (BRs) play crucial roles in plant development and also promote tolerance to a range of abiotic stresses. Although much has been learned about their roles in plant development, the mechanisms by which BRs control plant stress responses and regulate stress-responsive gene expression are not fully known. Since BR interacts with other plant hormones, it is likely that the stress tolerance conferring ability of BR lies in part in its interactions with other stress hormones.

**Results:**

Using a collection of Arabidopsis mutants that are either deficient in or insensitive to abscisic acid (ABA), ethylene (ET), jasmonic acid (JA) and salicylic acid (SA), we studied the effects of 24-epibrassinloide (EBR) on basic thermotolerance and salt tolerance of these mutants. The positive impact of EBR on thermotolerance in proportion to wild type was evident in all mutants studied, with the exception of the SA-insensitive *npr1-1 *mutant. EBR could rescue the ET-insensitive *ein2 *mutant from its hypersensitivity to salt stress-induced inhibition of seed germination, but remained ineffective in increasing the survival of *eto1-1 *(ET-overproducer) and *npr1-1 *seedlings on salt. The positive effect of EBR was significantly greater in the ABA-deficient *aba1*-*1 *mutant as compared to wild type, indicating that ABA masks BR effects in plant stress responses. Treatment with EBR increased expression of various hormone marker genes in both wild type and mutant seedlings, although to different levels.

**Conclusions:**

These results together indicate that the redox-sensitive protein NPR1 (NONEXPRESSOR OF PATHOGENESIS-RELATED GENES1), a master regulator of SA-mediated defense genes, is likely a critical component of EBR-mediated increase in thermotolerance and salt tolerance, but it is not required for EBR-mediated induction of *PR-1 *(*PATHOGENESIS-RELATED1*) gene expression; that BR exerts anti-stress effects independently as well as through interactions with other hormones; that ABA inhibits BR effects during stress; and that BR shares transcriptional targets with other hormones.

## Background

Brassinosteroids (BRs) are a group of plant steroidal hormones that regulate various aspects of plant growth and development, including cell elongation, photomorphogenesis, xylem differentiation, and seed germination [1], as well as adaptation to abiotic and biotic environmental stresses [2,3]. Molecular genetic studies of BR-deficient and BR-insensitive mutants have established an essential role for BRs in plant development and led to the identification and characterization of several BR signaling components [4,5]. A large number of BR-regulated genes have been identified by microarray studies; most of the known BR-regulated genes are associated with plant growth and development, such as cell wall modification, cytoskeleton formation, and hormone synthesis [4]. How BR regulates gene expression is currently understood for only a small proportion of genes. In the current model of BR-controlled gene expression, BR binding to BRI1, a plasma membrane localized leucine-rich repeat receptor-like kinase (LRR-RLK), induces association of BRI1 with its coreceptor BAK1, which enhances signaling output through reciprocal BRI1 transphosphorylation [4,5]. BRI1 binding to BR inactivates BIN2, a glycogen synthase kinase-3, and possibly activates the phosphatase BSU1. BIN2 negatively regulates transcription factors BZR1 and BES1 by phosphorylating them, while BSU1 positively regulates BR signaling by dephosphorylating BZR1 and BES1. Activated BZR1 and BES1 accumulate in the nucleus and directly bind to CGTG(T/C)G motif in the promoters of BR biosysnthesis genes *CPD *and *DWF4 *[6] and to E box sequence (CANNTG) in the *SAUR-ACI *promoter [7], respectively, to affect gene expression. The recent demonstrations that BES1 interacts with other transcription factors such as BIMs [7], MYB30, which acts as a positive regulator of the hypersensitive cell death response [8], and the jumonji (Jmj) domain-containing proteins ELF6 and REF6 that are involved in regulating flowering time [9], points to recruitment of different proteins by BES1 as one of the ways by which BR affects diverse biological processes.

The role of BRs in plant stress responses has been confirmed in several studies [10-13]. BR promotes tolerance in plants to a wide range of stresses, including heat, cold, drought and salinity, and this increase is generally correlated with higher expression of stress marker genes, such as *heat shock protein *(*hsp*) genes, *RD29A *and *ERD10 *[10,12], indicating that increased expression of stress-responsive genes is responsible, in part, for the higher stress tolerance in BR-treated plants. The mechanisms by which BR controls plant stress responses and regulates the expression of stress response genes are not known. Since different plant hormones can regulate similar physiological processes, and cross-talk between different hormones can occur at the level of hormone biosynthesis, signal transduction or gene expression [14], it was proposed that BR regulates plant stress responses via cross-talk with other hormones [2].

The plant growth regulators with documented roles in plant adaptation to abiotic and biotic stresses are abscisic acid (ABA), ethylene (ET), jasmonic acid (JA) and salicylic acid (SA). SA, JA and ET are important in defense against pathogen and pest attack [15], whereas ABA is a key molecule involved in salt and drought stress [16]. SA, ET, ABA and JA have also been linked to heat stress. Studies of hormone deficient and insensitive mutants have demonstrated the involvement of SA, ET and ABA in acquired thermotolerance of plants [17], and additionally for SA and JA, a role in basal thermotolerance of plants [18,19]. Although experimental evidence points to interactions of BR with auxin [20], gibberellin (GA) [21], ABA [22,23], ET [24,25] and JA [26], the relationship of BR with these hormones has been documented primarily in plant growth regulatory processes. Furthermore, with the exception of BR-auxin interaction, little is known in terms of genes how BR interacts with other hormones. Recent progress made towards understanding BR-auxin interaction can serve as a paradigm for how two hormones could interact at multi-levels. Auxin and BR share a number of target genes, many of which are involved in growth-related processes [20]. Since promoter regions in BR-responsive genes are enriched in Auxin Response Factor (ARF)-binding sites, and binding sites of BES1 are overrepresented in genes regulated by both hormones, regulatory elements in gene promoters represent a point of cross-talk between auxin and BR. Recently, the BR-regulated BIN2 kinase was demonstrated to phosphorylate ARF2, a member of the ARF family of transcriptional regulators, leading to loss of ARF2 DNA binding and repression activities [27]. Thus, in this model ARF2 links BR and auxin signaling pathways. In addition to gene coregulation, BR can also promote auxin transport [28], and optimal auxin action is dependent on BR levels [20].

The role of BR in plant responses to abiotic stress has become well established over the last decade, but there are very few reports indicating how BR interacts with other stress-related hormones and their signaling pathways in conferring stress tolerance. While there exists evidence to indicate that BR increases ET and JA levels under normal growth conditions [24,29], there appears to be only one report linking BR with increase in ABA levels in the lower plant *Chlorella vulgaris *under stress condition [30]. Recently it was demonstrated that ABA inhibits BR signaling through phosphorylation of BES1 [23]. Currently there are no studies at the genetic level as to how BR interacts with other hormones under stress conditions. Here we asked the question whether one or more stress-related hormones, such as ABA, ET, JA or SA, have a major role in BR-mediated stress tolerance. Arabidopsis mutants with either disrupted or enhanced hormone pathways were tested for phenotypes and gene expression in response to BR under high temperature and high salt conditions. Our results suggest that in Arabidopsis the NONEXPRESSOR OF PATHOGENESIS-RELATED GENES1 (NPR1) is likely a critical component of BR-mediated effects on thermotolerance and salt tolerance, that BR exerts anti-stress effects both independently as well as through interactions with other hormones, ABA inhibits BR effects during heat stress, and that BR shares transcriptional targets with other hormones.

## Results

### EBR effects on basal thermotolerance in different hormone genotypes

**Figure 1:**
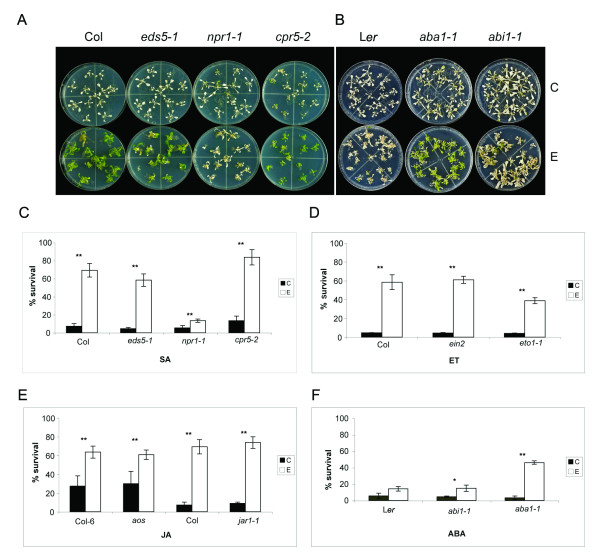
**Effect of EBR treatment on basal thermotolerance of Arabidopsis WT and SA, ET, JA and ABA mutant seedlings**. **(A and B) **WT and mutant seedlings grown on a nutrient medium in the absence (C) or presence of 1 μM EBR (E) were exposed to 43°C for 4 h. Photographs of the seedlings were taken after recovery at 22°C for 7 days. **(C-F) **Proportion of WT and mutant seedlings surviving the lethal heat stress (43°C for 4 h). Seedlings grown and treated as described in methods were scored as dead or surviving after 7 days of recovery at 22°C. WT controls were L*er *for *abi1-1 *and *aba 1-1*, Col-6 for *aos*, and Col for all other mutants. Data shown are average of three replicates. Error bars represent standard error (SE) of mean for three replicates. Asterisks above the histograms indicate the significance of differences between untreated and EBR-treated genotypes as analyzed by Student's *t*-test (**p < 0.01; *p < 0.05).

We have previously demonstrated that EBR enhances the basic thermotolerance of *Brassica napus*, tomato [10,11] and Arabidopsis seedlings [12]. Since the effects of EBR on stress tolerance are most pronounced when seedlings are grown in the presence of EBR (long-term treatment), we postulated the involvement of other phytohormones in this process [2]. Several hormone pathways, such as of ABA, ET, SA and JA, have been linked with one or more environmental stresses, including heat stress (HS). We therefore evaluated the effects of EBR on thermotolerance in a subset of Arabidopsis hormone mutants altered in either biosynthesis or signaling of these phytohormones (Table 1). We first studied the effects of EBR on basal thermotolerance in SA genotypes *npr1-1 *(defective in signalling for SA-mediated systemic acquired resistance (SAR), but not all SA responses), *eds5-1 *(defective in SA synthesis), and *cpr5-2 *(elevated SA levels, enhanced levels of *PR*, and constitutive expression of both NPR1-dependent and NPR1-independent SAR pathway) by exposing 21-day-old seedlings to 43°C for 4 h, allowing them to recover at 22°C for 7 days and then scoring for dead and surviving seedlings. WT, *eds5-1*, *npr1-1 *and *cpr5-2 *seedlings grown in the absence of EBR had average survival rates of 7.5, 4.5, 5.5 and 13.5%, respectively, and those grown in the presence of EBR had survival rates of 69.3, 58.3, 13.4 and 83%, respectively (Figure 1A and 1C). From these results it is clear that the survival rates of WT and different SA genotypes were significantly increased by EBR treatment, but the increase was considerably less in case of *npr1-1 *as compared to other genotypes within this group. Photographs of all genotypes grown in the presence or absence of EBR under no-stress conditions can be seen in additional file 1: Pictures of 21-day-old seedlings.

**Table 1:** Mutant alleles used in the study with the description of corresponding genes and mutant phenotypes

Category	Locus (AGI)	Gene	Description	Mutant allele used	References
SA synthesis Defective	AT4G39030	*EDS5*	member of the MATE-transporter family; mutants do not accumulate SA after pathogen inoculation and are hypersusceptible to pathogen infection.	*eds5-1*	[73,74]
SA-insensitive	AT1G64280	*NPR1*	similar to the transcription factor inhibitor I kappa B, and contains ankyrin repeats; key regulator of SA-mediated systemic acquired resistance (SAR) pathway; mutants are SA-insensitive and hypersusceptible to pathogen infection.	*npr1-1*	[43,75,76]
High SA levels	AT5G64930	*CPR5*	regulator of expression of pathogenesis-related (*PR*) genes; participates in signal transduction pathways involved in plant defense; mutants exhibit increased SA levels and constitutive expression of *PR *genes.	*cpr5-2*	[63]
ET-insensitive	AT5G03280	*EIN2*	NRAMP metal transporter family; involved in ET signal transduction; mutants are ET-insensitive.	*ein2*	[77]
High ET levels	AT3G51770	*ETO1*	encodes a negative regulator of 1-aminocyclopropane-1-carboxylic acid synthase5(ACS5), which catalyzes the rate-limiting step in ET biosynthesis; mutations elevate ET biosynthesis by affecting the posttranscriptional regulation of ACS.	*eto1-1*	[77]
JA-deficient	AT5G42650	*AOS*	encodes a member of the cytochrome p450 CYP74 gene family that functions as an allene oxide synthase; catalyzes dehydration of the hydroperoxide to an unstable allene oxide in the JA biosynthetic pathway; mutants are JA-deficient.	*aos*	[78]
JA-insensitive	AT2G46370	*JAR1*	encodes cytoplasmic localized phytochrome A signaling component protein similar to the GH3 family of proteins; loss of function mutants are defective in a variety of responses to JA.	*jar1-1*	[79]
ABA-deficient	AT5G67030	*ABA1*	encodes zeaxanthin epoxidase gene that functions in first step of ABA biosynthesis; mutants are ABA-deficient.	*aba1-1*	[80]
ABA-insensitive	AT4G26080	*ABI1*	Protein phosphatase 2C; involved in ABA signal transduction; mutants are ABA-insensitive.	*abi1-1*	[81]

For genotypes related to ethylene, EBR increased survival rates of WT, *ein2 *(ET-insensitive), and *eto1-1 *(ET-overproducer) seedlings to significant levels as compared to seedlings with no treatment (Figure 1D).

The JA mutants *aos *(JA-deficient) and *jar1-1 *(defective in JA response) belong to different backgrounds; *jar1-1 *is in Arabidopsis ecotype Columbia (Col), whereas *aos *is in Col-6 background. EBR increased survival rates of *aos *and *jar1-1 *seedlings to amounts similar to corresponding WT seedlings (Figure 1E).

The ABA mutants *aba1-1 *(ABA-deficient) and *abi1-1 *(ABA-insensitive) are from the Landsberg *erecta *(L*er*) background; hence, WT L*er *was used for comparison with these mutants. Under the conditions used, EBR was less effective in WT L*er *as compared to WT Col (about 2.5-fold increase in L*er vs *9-fold increase in Col in survival rates in response to EBR) (Figure 1E and 1F). Since ABA has been linked with heat tolerance [17,31], we expected ABA mutants to be less thermotolerant than WT even in the presence of EBR. Contrary to our expectation we found that the effect of EBR was most distinct in *aba1-1 *(survival rate of 43.3%) as compared to WT (14.3%) and *abi1-1 *(15%) seedlings (Figure 1B and 1F). These results suggest that ABA masks BR effects on the HS response pathway of WT Arabidopsis seedlings.

From the survival data represented in Figure 1 it is clear that when EBR effect in any hormone genotype is viewed in reference to the effect on the corresponding WT, EBR could increase the basal thermotolerance of all hormone genotypes, but its effect was minimal in *npr1-1*. Since the SA-deficient *NahG *transgenic line [32] and the JA response defective *coi1 *mutant [33] could not be obtained for this study, we cannot yet conclude that SA and JA are dispensable for BR-mediated increase in thermotolerance. However, from the collection of mutants used here it would appear that BR can exert anti-stress effects that are independent of ABA, ET, JA and SA, at least to some extent. The dependency of BR on NPR1 in mediating stress tolerance is a first time observation made in this study. Whether BR modulates NPR1 activity via SA or BR pathway or both remains to be determined.

### EBR effects on oxidative damage in hormone mutants

**Figure 2:**
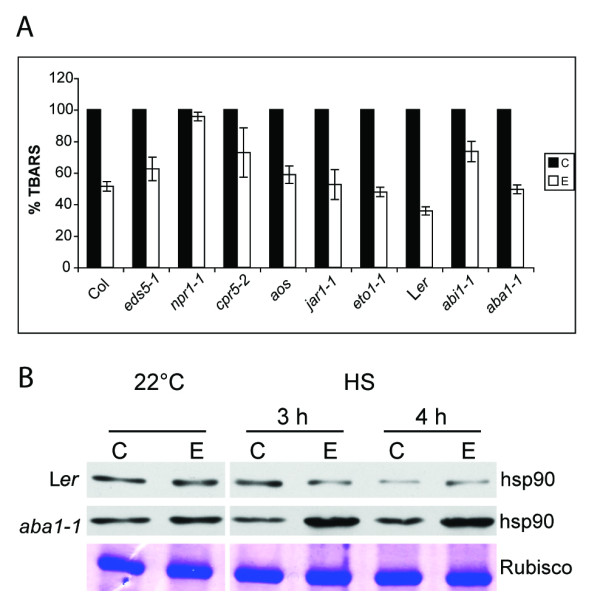
**Effect of EBR treatment on heat induced-oxidative damage and hsp90 accumulation in WT and mutant seedlings**. **(A) **Percent TBARS was measured for untreated (C) and EBR-treated (E) WT and mutant seedlings exposed to 43°C for 3 h and allowed to recover at 22°C for 2 days. Data shown are average of three replicates. Error bars represent SE of mean for three replicates. The differences between the (C) and (E) values for *npr1-1 *and *cpr5-2 *were not significant, but for all other genotypes the differences were significant to *p *< 0.02. **(B) **Total protein was isolated from WT (L*er*) and *aba1-1 *seedlings grown in the absence (C) or presence (E) of EBR at 22°C and either maintained at 22°C or exposed to 43°C for 3 h and 4 h (HS). Samples were analyzed by Western blotting using an anti-hsp90 antibody. Coomassie blue staining of ribulose-1, 5-biphosphate carboxylase/oxygenease (rubisco) was used as loading control.

Heat stress produces oxidative damage, which as a result of lipid peroxidation leads to the production of thiobarbituric acid reactive substances (TBARS) [17,34]. To complement the results of the HS phenotype of seedlings, oxidative damage levels were assessed in untreated and EBR-treated seedlings. Measurements of TBARS in time course experiments determined that maximum oxidative damage occurred during recovery from HS (data not shown). Therefore, seedlings exposed to HS and then allowed to recover for 2 days at 22°C were used for TBARS analysis. With the exception of *npr1-1 *and *cpr5-2*, EBR treatment reduced the levels of oxidative damage in WT and other mutant seedlings as compared to their untreated counterparts (Figure 2A). WT Col and L*er *exhibited 45% and 60% reduction in TBARS production, respectively, while the mutant seedlings showed 25-50% less TBARS, in response to EBR treatment. Consistent with its lower survival rate (Figure 1), the *npr1-1 *mutant showed an insignificant 4.5% reduction in TBARS production in response to EBR treatment (Figure 2A). However, in contrast to the relatively higher survival of *cpr5-2 *seedlings, the reduction in oxidative damage in these seedlings in response to EBR measured only 25% relative to no treatment. Overall, these results demonstrated that EBR treatment can reduce oxidative damage during HS and that this effect is not critically dependent on any one hormone in question, although a functional NPR1 protein appears to be required for a complete effect of EBR on thermotolerance of seedlings.

### EBR induces higher accumulation of hsp90 in *aba1-1*

We have found that EBR treatment leads to significant increases in the levels of hsps during HS in *B. napus *[10,11], but the effect of EBR on hsp levels in Arabidopsis is subtle [12]. We wished to see how EBR would affect the accumulation of hsp90 in various mutant seedlings in the absence of HS (22°C) and in response to HS (3 h and 4 h exposure to 43°C). With the exception of *aba1-1 *(Figure 2B), no significant differences in the steady state levels of hsp90 were observed between EBR-treated and untreated mutant seedlings, including *npr1-1 *(data not shown). EBR-treated *aba1-1 *seedlings accumulated approximately 3- and 2.5-fold higher levels of hsp90 at 3 and 4 h of HS, respectively, as compared to untreated *aba1-1 *seedlings (Figure 2B). By contrast, EBR-treated WT seedlings showed a maximum of 1.3-fold increase in hsp90 levels at 4 h of HS as compared to untreated seedlings. The fold-change values are average of three different experiments, which consistently produced the same pattern of protein accumulation. Thus, with respect to higher survival following HS and greater accumulation of hsp90 during HS, EBR produced most distinct effects in the ABA-deficient *aba1-1 *mutant. These results reinforce the idea that ABA suppresses BR effects in WT seedlings. Although convincing evidence for antagonism between BR and ABA in plant growth regulatory processes, such as germination has been provided before [22,23], the demonstration at the genetic and molecular levels of an antagonistic relationship between the two hormones in plant stress response is new.

### EBR upregulates the expression of SA, JA/ET and ABA-response genes in both WT and corresponding mutants

Plant hormone responses in Arabidopsis have been correlated with the expression of hormone-specific marker genes. We studied the expression of few such genes that are well documented to be induced by SA, JA/ET or ABA, both before and after treatment with EBR. *PR-1*, transcription factor *WRKY70 *and *WAK1 *are known to be regulated primarily by SA [35-37]; *PDF1.2*, *LOX2 *and *HEL *by JA/ET [38-40]; and *RD22 *and *LTP4 *by ABA [41,42].

**Figure 3:**
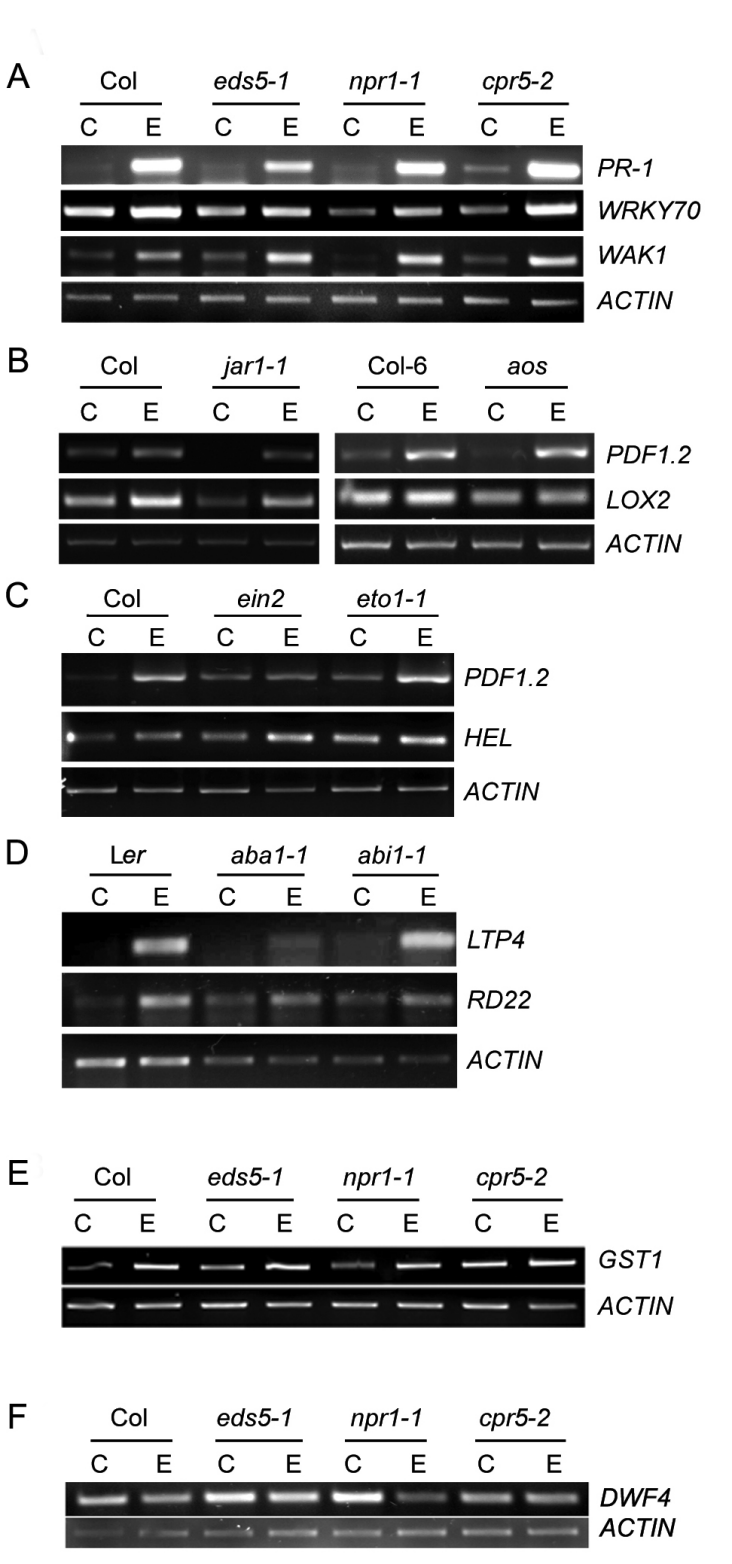
**Effect of EBR on the expression of SA, JA/ET and ABA response genes**. Total RNA was isolated from WT and mutant seedlings grown in the absence (C) or presence (E) of EBR at 22°C, and transcript levels were analyzed by RT-PCR. Actin was included as a control for constitutive expression. **(A-D) **Expression profiles of *PR-1, WRKY70*, and *WAK1 *in WT and SA-related genotypes; *PDF1.2 *and *LOX2 *in WT and JA mutants; *PDF1.2 *and *HEL *in ET-related genotypes; *LTP4 *and *RD22 *in ABA mutants. **(E) **Expression profile of the stress-responsive *GST1 *in WT (Col) and SA genotypes. **(F) **Down-regulation by EBR of *DWF4 *in WT (Col) and SA-insensitive *npr1-1 *mutant.

The steady-state levels of *PR-1*, *WRKY70 *and *WAK1 *transcripts were elevated by EBR in WT and SA-related genotypes, including *npr1-1*, albeit at different levels (Figure 3A). The *npr1-1 *genotype is defective in the expression of *PR *genes in response to SA [43]. Full-scale induction of *WRKY70 *and *WAK1 *by SA also requires a functional NPR1 [36,37]. Our results clearly indicate that EBR can mediate induction of *PR-1*, *WRKY70 *and *WAK1 *to levels seen in Figure 3A in an NPR1-independent manner.

EBR treatment also enhanced the expression of the JA/ET marker gene *PDF1.2 *in WT, *aos*, *jar1-1 *and *eto1-1 *backgrounds, but not to the same extent in *ein2 *(Figure 3B and 3C). The effect of EBR on *LOX2 *expression was distinct in Col and *jar1-1 *backgrounds, but not in Col-6 and *aos *backgrounds. Increase in the expression of the *HEL *gene by EBR was only slight. The ABA-responsive *LTP4 *showed dramatic induction by EBR in WT and *abi1-1 *background (Figure 3D), but not in *aba1-1*. The transcript levels of the ABA-marker gene *RD22 *were upregulated by EBR only slightly in *aba1-1 *and *abi1-1 *mutant seedlings, but significantly in WT (Figure 3D), indicating interaction between ABA and BR in affecting gene expression.

To determine the interaction of EBR with SA, JA/ET and ABA in the regulation of *GST1*, a gene common to abiotic stress and defense pathways [44], we compared its transcript levels in untreated and EBR-treated WT and mutant seedlings. EBR enhanced *GST1 *transcript levels in SA (Figure 3E), as well as in all other hormone genotypes studied (data not shown). As would be expected, *cpr5-2 *had the highest expression of *GST1 *even in the absence of EBR.

Exogenous BR negatively regulates the BR biosynthetic gene *DWF4 *[6]. To ensure that transport, perception and signaling of BR is intact in *npr1-1*, which was most inert to EBR effects, *DWF4 *expression was determined in WT and *npr1-1*. The fact that *DWF4 *levels were reduced in EBR-treated WT and *npr1-1 *seedlings as compared to untreated seedlings (Figure 3F) suggests that the BR pathway is intact in *npr1-1*.

Taken together, these results demonstrate that most, if not all, of the SA, JA/ET and ABA-responsive genes tested in the present study are also upregulated by BR both in WT and mutant backgrounds, albeit to different levels. These results point to overlapping gene targets of ABA, JA/ET or SA and BR, as well as to hormone interactions controlling the final output.

### Upregulation of a subset of genes by EBR is likely via a BR-regulated pathway

**Figure 4:**
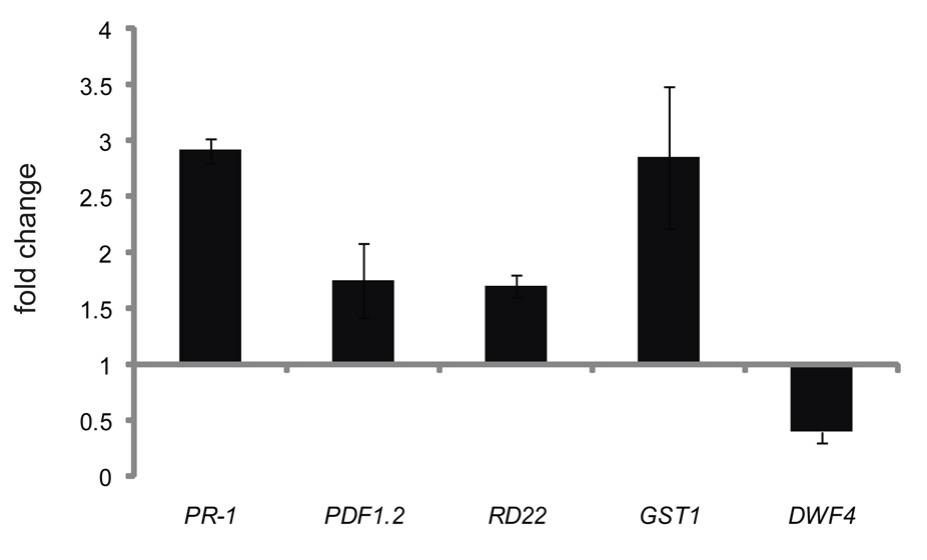
**Effect of EBR on the expression of SA, JA/ET and ABA response genes after short-term treatment with EBR**. WT (Col) seedlings grown for 21 days in the absence of EBR were treated with 1 μM EBR (E) or 0.01% ethanol (C) for 7 h. Transcript levels of *PR-1*, *PDF1.2*, *RD22*, *GST1*, *DWF4 *and *UBQ10 *were analyzed by qRT-PCR. *DWF4 *(down-regulation) was used as a control for BR-regulated gene expression.

Since the effect of EBR on thermotolerance is best seen when plants are grown in the presence of EBR for 2-3 weeks, the gene expression studies leading to Figure 3 were also conducted in plants receiving long-term exposure to EBR. To determine whether the JA/ET and ABA-responsive genes also respond to a short-term EBR treatment, we studied the expression of a subset of genes by qRT-PCR in response to a 7 h treatment with EBR. It is to be noted that BR effects on gene expression can take as long as 18-48 hrs [45,46]. Similar to the results of long-term treatment, the 7 h short-term treatment with EBR enhanced the transcript levels of *PR-1 *(~2.9-fold), *PDF1.2 *(~1.8-fold), *RD22 *(~1.7-fold) and *GST1 *(~2.9-fold), suggesting that changes in the expression of these genes likely occur via a BR-regulated pathway (Figure 4). Further studies are required to confirm this idea. Downregulation of *DWF4 *by exogenous BR was used as an experimental control (Figure 4).

### EBR rescues hypersensitivity of *ein2 *to inhibition of germination by salt stress

**Figure 5:**
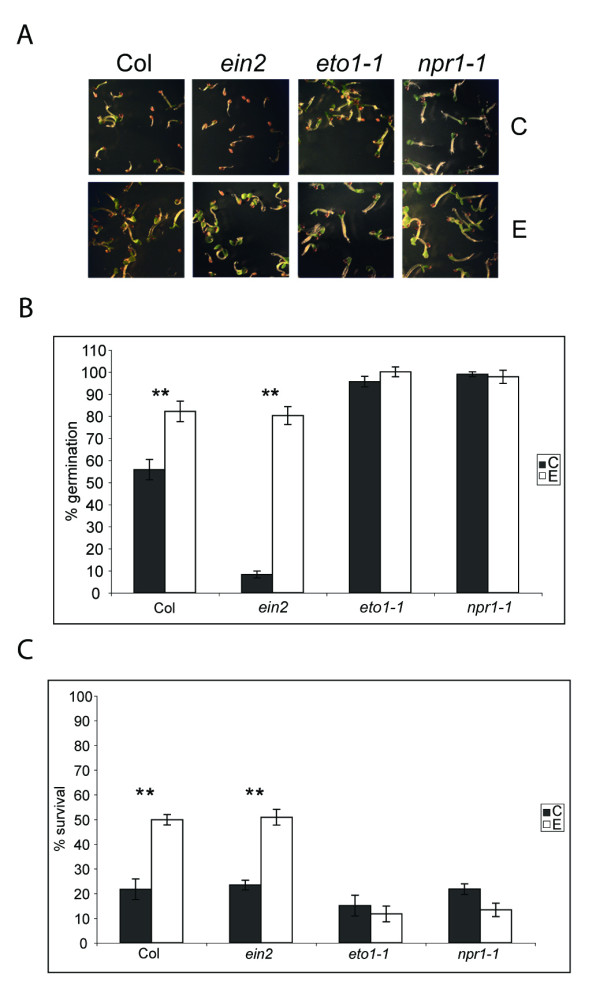
**EBR effects on inhibition of germination by NaCl in WT and mutant seedlings**. **(A) **WT (Col), *ein2*, *eto1-1 *and *npr1-1 *were allowed to germinate on a nutrient medium containing 150 mM NaCl in the absence (C) or presence of 1 μM EBR (E). Photographs of the seedlings were taken 3 days after imbibition. **(B) **Percentage of seeds germinated was calculated by counting the number of seedlings with emerged cotyledons at 3 days after imbibition. **(C) **Percentage of seedlings surviving on 150 mM NaCl was calculated by counting the number of seedlings that showed true leaves and green colour at 20 days after imbibition. All experiments were performed in triplicates with n > 30. Error bars represent SE of mean for three replicates. Asterisks above the histograms indicate the significance of differences between untreated and EBR-treated genotypes as analyzed by Student's *t*-test (**p < 0.01).

We have previously shown that EBR helps to overcome salt stress-induced inhibition of seed germination in *B. napus *[12]. More recently it was demonstrated that BR-deficient and BR signaling-defective mutants are more inhibited in germination on salt while a BRI1 overexpressing transgenic line is more resistant to salt than WT [23]. In this study, we first screened *npr1-1, ein2, eto1-1, jar1-1 *and *abi1-1 *mutants for their germination efficiency on 150 mM NaCl. As noted before with *ein2-5 *[47], *ein2 *was more sensitive to NaCl-mediated inhibition of germination as compared to WT (Figure 5A and 5B). By contrast, *eto1-1, npr1-1, jar1-1 *and *abi1-1 *were less sensitive than WT (data not shown). Thus, to study the effects of EBR on salt stress we selected *ein2 *because of its hypersensitivity to salt stress, and *eto1-1 *and *npr1-1 *because of their relatively higher susceptibility to HS even after EBR treatment. Seeds were allowed to germinate on 150 mM NaCl in the presence or absence of EBR, and seedlings with emerged cotyledons were scored after 3 days. Inhibition of germination of *ein2 *seeds by salt was significantly reduced in the presence of EBR (Figure 5A and 5B). WT seeds showed germination rates of 55% and > 80% on 150 mM NaCl in the absence and presence of EBR, respectively, while *ein2 *seeds had germination rates of ~10% and 80% under the same conditions (Figure 5B). Seeds of *eto1-1 *and *npr1-1 *germinated at similar rates (> 90%) on 150 mM NaCl in the absence or presence of EBR (Figure 5B). However, despite the good germination efficiency on salt, the average survival rates of *eto1-1 *and *npr1-1 *on salt were only 15 and 22%, respectively (close to WT), but unlike WT, survival of the mutant seedlings could not be rescued by EBR treatment (Figure 5C). These results indicate that NPR1 has a role in salt stress and that BR effects on seedling survival under salt stress require a functional NPR1. Phenotypes of WT, *ein2, eto1-1 *and *npr1-1 *seedlings grown in the presence or absence of EBR under no-salt conditions can be seen in additional file 2: Pictures of 3-day-old seedlings.

## Discussion

Feeding BR through roots (long-term treatment) not only rescues growth defects of BR-deficient mutants [48-50], but also leads to increased stress tolerance in WT seedlings [10-12]. Furthermore, overexpression of BR biosynthesis genes leads to increased vegetative and seed yield in transgenic seedlings [51]. In each case it is rationalized that BR-mediated outcomes likely involve both direct and indirect effects of BR. In the present study we focused on understanding BR interactions with other stress hormones in mediating increase in stress tolerance mainly because 1) BR is known to interact with other plant hormones in regulating plant developmental processes, and 2) multiple hormone signaling pathways play a role in acquisition of stress tolerance. We evaluated a subset of signaling, biosynthetic and constitutively active mutants of ABA, ET, JA and SA for thermo and salt tolerance in untreated and BR-treated states to assess the importance of these hormones in BR-mediated increase in stress tolerance of Arabidopsis seedlings. Here we demonstrate that NPR1, a protein well recognized for its role in SA-mediated SAR and cross-talk inhibition of JA-mediated defense responses, also has a role in BR-mediated stress tolerance.

### Thermotolerance defects of hormone mutants and BR effects

SA, and more recently JA, have been linked with thermotolerance. In case of SA genotypes, it is known that *npr1-1 *is compromised in basal thermotolerance, while *cpr5-2 *has greater thermotolerance than WT [17,18]. Recently it was demonstrated that a JAR1-dependent pathway is also required for basal thermotolerance [19]. Using a collection of genotypes with basal thermotolerance either lower or higher than WT, we found that EBR treatment could significantly increase the basic thermotolerance of these genotypes and that this increase was comparable to the increase in WT. An exception to this result within the SA, JA and ET genotypes was *npr1-1*, indicating that a functional NPR1 is required for full manifestation of BR's effects. Although collectively our data seems to suggest that BR is not critically dependent on SA levels and JA signaling for its antistress effects, further confirmation is required with genotypes such as *Nahg *and *coi1 *to make an unequivocal claim. Even if BR works to some extent independently of other hormones in conferring heat tolerance, the mere fact that ABA, BR, ET, JA and SA all play a role in thermotolerance of Arabidopsis plants suggests that there must be some redundant and some specific events in the mechanisms by which these hormones produce their effects. With the exception of BR where some information has been obtained [10-12], molecular changes mediated by ABA, ET, JA and SA that lead to thermotolerance are largely unknown. In case of ethylene, the Ethylene Response Factor Protein, JERF3, has been demonstrated to activate the expression of oxidative genes, resulting in decreased accumulation of ROS and, in turn, enhanced adaptation to drought, freezing, and salt in tobacco [52]. A similar role for JERF3 can be envisioned in response to HS. A number of ABA-regulated genes have been implicated in drought tolerance [16]. Recent functional characterization of the ABA-regulated ERD10 and ERD14 indicated that these proteins could prevent the heat-induced aggregation and/or inactivation of various enzyme substrates [53]. Thus, induction of genes functionally similar to molecular chaperones by ABA during HS may help combat the denaturing stress effects of HS.

Previous studies involving treatment with exogenous ABA [31], *aba *and *abi *mutants [17], and high ABA producing lines [54], have demonstrated the positive effects of ABA on thermotolerance. Under our experimental conditions the differences in the survival rates of untreated WT and *aba1-1 *and *abi1-1 *mutant seedlings were not striking, but the most pronounced effects of EBR with respect to survival within this set of plants was seen in *aba1-1 *seedlings (Figure 1F), indicating that endogenous ABA levels suppress BR effects. This notion is supported further by higher accumulation of hsp90, a representative of the hsp families of proteins that are known markers of thermotolerance, in *aba1-1 *seedlings as compared to WT (Figure 2B). It should be noted that neither SA, nor ABA or BR mutants are compromised in hsp accumulation [12,17] [present study]. We have previously demonstrated that treatment with exogenous EBR can significantly increase hsp accumulation in *B. napus *[10,11], but that this effect in Arabidopsis is subtle [12]. Thus, the clear enhancement of hsp accumulation in response to EBR during HS in *aba1-1*, but not in WT (Figure 2B), confirms that endogenous ABA levels suppress BR effects in WT even under stress conditions. While this work was under preparation, a study describing ABA inhibition of BR signaling was reported whereby it was shown that ABA increases the expression levels of BR biosynthesis gene *DWF4 *and *CPD *to a greater extent in *aba1 *than in WT, reinforcing the idea that ABA inhibits BR signaling in WT and that this inhibition is relieved in the *aba1 *background [23].

**Figure 6:**
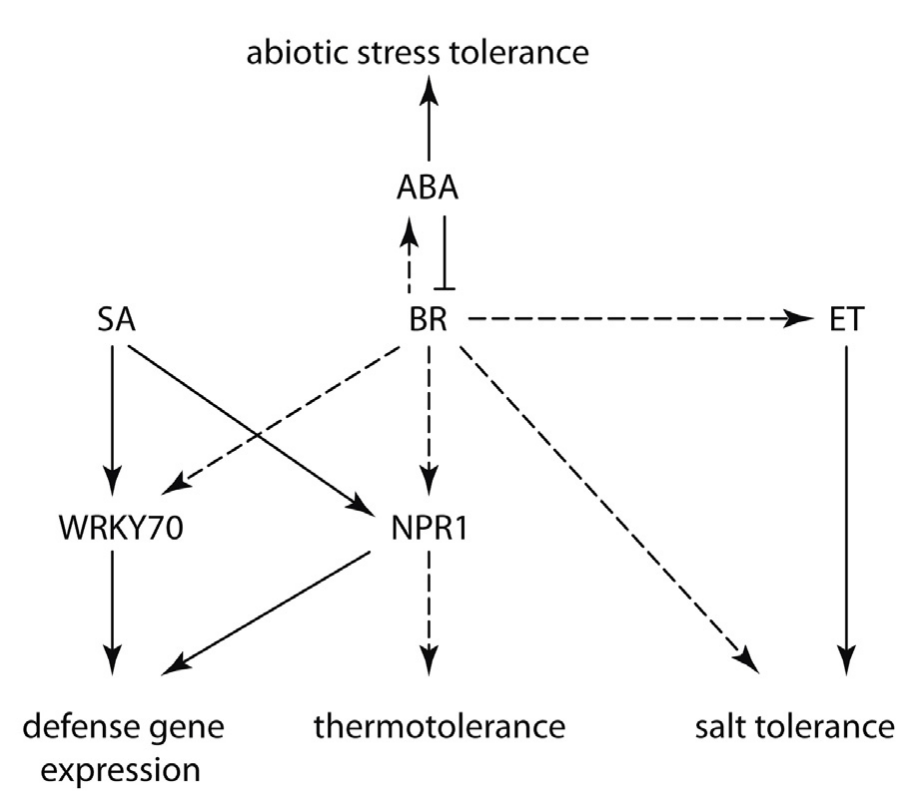
**Proposed model for interaction of BR with SA, ABA and ET in mediating stress tolerance**. BR positively regulates SA pathway components NPR1 and WRKY70 to mediate thermotolerance and defense gene expression, respectively. ABA suppresses BR effects on thermotolerance, but BR may enhance ABA responses under certain conditions by increasing ABA levels. BR can enhance salt tolerance through an ET-independent pathway or ET-dependent pathway. The dashed arrows represent BR responses, while solid arrows indicate other hormone pathways.

Although BR and ABA display an antagonistic relationship in some physiological responses, we observed in a microarray experiment that several ABA-responsive genes and one ABA biosynthesis gene were upregulated by EBR and HS [P. Krishna and coworkers, unpublished results]. These results suggest that ABA levels rise in response to HS in Arabidopsis and that this increase may be further augmented by BR treatment. Indeed, ABA content has been reported to increase in pea leaves in response to HS [55] and in response to BR under HS in *C*. *vulgaris *[30]. Based on these results it can be speculated that BR augments ABA levels and ABA-related effects during HS (Figure 6), but it is only when ABA levels are compromised that the BR effects of enhancing stress tolerance become apparent.

### NPR1, stress tolerance and BR effects

Although *npr1-1 *seedlings have been shown to be defective in basal thermotolerance, the heat sensitivity of this genotype is not dramatically lower than WT [17-19]. However, in our study the mere 2.4-fold increase in percent survival of *npr1-1 *in response to EBR treatment as compared to the 9-fold increase in WT Col following HS (Figure 1), nearly no change in oxidative damage levels in *npr1-1 *in response to EBR (Figure 2A), and the lack of EBR effect in increasing survival of *npr1-1 *seedlings on salt (Figure 5B), explicitly indicate that a functional NPR1 is required for the manifestation of BR effects on seedling stress tolerance. Two questions arise from these observations; 1) what would be the function of NPR1 during abiotic stress conditions, and 2) how could NPR1 integrate in the BR pathway?

NPR1 is a redox-controlled transcriptional cofactor, which is key to development of SAR and critical for modulating cross-talk between SA and JA signaling [56]. In the absence of stress, NPR1 is maintained in a large complex consisting of intermolecular disulfide bonded oligomers, which upon stress are reduced to an active monomeric state [57]. The monomeric form interacts with TGA-bZIP transcription factors and activates defense gene expression. ROS is a common signal in plant stress responses to both abiotic and biotic stresses [58,59]. The notion that ROS could be a signal for the HS response is derived from studies of activation of heat shock transcription factors (Hsfs). The eukaryotic Hsf1 multimerizes and binds to DNA upon either heating or oxidation with H_2_O_2 _[60]. More direct evidence showing that endogenous ROS production is necessary for induction of the HS response comes from the observation that a dominant negative allele of Rac1, the small GTPase necessary for the activation of ROS production by membrane-bound NADPH oxidase [61], inhibits the stress-induced activation of Hsf1 [62]. Thus, it is highly likely that ROS production during HS also activates NPR1, leading to gene expression changes critical for thermotolerance. Future studies directed at global gene expression analysis in WT and *npr1 *mutant in response to HS and BR as separate and combined treatments should indicate NPR1-dependent molecular changes and help clarify the role of NPR1 in BR-mediated increase in thermotolerance.

The mechanism for how BR could function with NPR1, it is speculated that ROS-activated NPR1 monomers may bind a BR-activated regulator to affect stress-responsive gene expression critical to the survival of seedlings under stress conditions. It is unlikely that NPR1 controls BR signaling via BIN2 and BZR1 given that downregulation of DWF4 in *npr1-1 *was unaffected (Figure 3F).

### Oxidative stress state in *cpr5 *and BR effects

The *cpr *(CONSTITUTIVE EXPRESSOR OF *PR *GENES) mutants are characterized with increased concentrations of SA, constitutive expression of the *PR *genes, and enhanced resistance to pathogens [63,64]. The *cpr *mutants are in a state of high-cellular oxidative stress state as compared to WT. For example, *cpr1 *plants exhibited greater oxidative damage than WT under both normal growth (23°C) and chilling (5°C) conditions [65], and molecular changes in *cpr5 *such as increased expression of several genes in the ROS gene network, including GSTs, indicate that the cellular redox balance in this mutant is deregulated [66]. We also observed higher levels of *GST1 *transcript in *cpr5-2 *as compared to WT (Figure 3B). The high-cellular oxidative stress state of the *cpr *mutants, combined with the constitutively activated SA and JA/ET-mediated pathways [64,67], may trigger not only defense responses against pathogens but also abiotic stress response pathways, leading to greater survival of *cpr5-2 *seedlings than WT in response to HS (Figure 1B). EBR treatment further enhanced the survival rates of *cpr5-2 *seedlings exposed to HS (Figure 1B), but had negligible effect on the oxidative damage levels in this mutant (Figure 2A), presumably due to the inherent oxidative state of the mutant. This result does not preclude other positive effects of EBR on *cpr5-2 *physiology, leading to increased stress tolerance.

### BR induces expression of other hormone marker genes

Upregulation of *PR-1 *by BR in *eds5-1 *and *npr1-1 *backgrounds (Figure 3A), as well as in short-term treatment (Figure 4), suggests that BR directly mediates expression of this gene. Interestingly, we found *WRKY70*, a transcription factor acting downstream of NPR1 and involved in the expression of SA-induced *PR *genes [36], to also be upregulated by BR in different SA genotypes (Figure 3A). Thus, in addition to NPR1, *WRKY70 *may be a potential point of cross-talk between SA and BR via which BR may induce a subset of SA-responsive genes. Hormone response data obtained using AtGenExpress Visualization Tool (AVT) http://jsp.weigelworld.org/expviz/expviz.jsp indicate that SA and BR are the two most potent inducers of *WRKY70 *expression (data not shown), which reinforces the idea that *WRKY70 *is an overlapping transcriptional target of both SA and BR. Such a scenario could explain, in part, how BR enhances plant resistance against pathogen infection [2].

BR could increase the expression of *PDF1.2 *in JA mutants, but not to the same extent in the ET-insensitive mutant *ein2 *(Figure 3B and 3C). Thus, at present we favour the possibility that BR effects on *PDF1.2 *expression are mediated via the ET pathway. It should be noted that *PR-1 *and *PDF1.2 *genes are constitutively expressed in *cpr *and *ssi1 *mutants of Arabidopsis in an NPR1-independent, and SA, JA and ET-dependent manner [67,68], providing precedent for the notion that more than one pathway governs the expression of these genes. The increase in the transcript levels of the ABA-responsive *RD22 *gene and the stress-induced *GST1 *gene in WT and hormone mutants by EBR, including short-term treatment of WT (Figure 4), suggests that these genes may also be primary targets of BR. Taken together, our data indicate that BR has regulatory inputs into the expression of other hormone-responsive genes, which may result from the action of the BR pathway either on the promoters of these genes, or on the regulation of a hormone-signaling component or the biosynthesis of another hormone (Figure 6).

### BR effects on inhibition of seed germination by salt

We have previously shown in *B. napus *that EBR helps to overcome inhibition of seed germination by salt [12]. To study the involvement of BR and other hormones in salt tolerance, we screened WT, *npr1-1*, *jar1-1*, *abi1-1*, *ein2 *and *eto1-1 *seeds for germination on 150 mM salt. The *ein2-5 *mutant has been found to be hypersensitive to salt [47] and *ein2-1 *displayed sensitivity to heat and osmotic stress [69]. Clearly, EIN2 is an important node for interaction of stress and hormonal signaling pathways. The fact that EBR could rescue hypersensitivity of *ein2 *to salt (Figure 5A and 5B) and increase survival rates of *ein2 *seedlings following HS (Figure 1) with effects paralleling those in WT, unambiguously indicates that BR can bypass ET signaling in conferring stress tolerance in Arabidopsis.

We included *npr1-1 *and *eto1-1 *in the salt stress study due to the hypersensitivity of these genotypes to HS even in presence of BR. Interestingly, while both *npr1-1 *and *eto1-1 *were insensitive to salt during germination, both genotypes had survival rates comparable with those of WT and *ein2 *in the absence of EBR, and notably lower survival rates than WT and *ein2 *in the presence of EBR (Figure 5C). These results further endorse a crucial requirement of functional NPR1 in BR-mediated increase in stress tolerance and suggest further explorations of the roles of NPR1 in abiotic stress. The possibility that NPR1 mediates defense responses against abiotic stresses has been suggested in two recent reports [70,71].

## Conclusions

In summary we have demonstrated that 1) BR-mediated increase in stress tolerance is integrated with other hormone pathways, 2) NPR1 appears to be a critical component of BR-mediated effects on thermo and salt tolerance, 3) ABA inhibits BR effects in abiotic stress responses, 4) the effect of BR in overcoming inhibition of germination by salt is independent of EIN2, and 5) several hormone-responsive genes are also BR-responsive. Overall, these findings point to possible cross-talk of BR with SA, ET and ABA signaling pathways in mediating stress responses, as depicted in Figure 6.

## Methods

### Plant material and growth conditions

The *eds5-1 *(CS3735)*, npr1-1 *(CS3726)*, cpr5-2 *(CS3770)*, ein2 *(CS8844), *eto1-1 *(CS3072), *aos *(CS6149), and *jar1-1 *mutants belong to the Col background, while the *aba1-1 *(CS21) and *abi1-1 *(CS22) mutants are in the L*er *background. Accordingly, the WT Col and L*er *were used as controls in the experiments. The *aos *mutant is a T-DNA knock-out line derived from Col-6, hence its parental line (CS8155) was used as control. The *jar1-1 *mutant was a kind donation from Dr. Pradeep Kachroo (University of Kentucky, Lexington, KY, USA). All other mutants were obtained from the Arabidopsis Biological Resource Center (ABRC).

Seedlings were grown essentially as described by Kagale et al. [12]. Seeds were surface sterilized and plated on 1× Murashige and Skoog medium (Sigma, St. Louis) supplemented with B5 vitamins, 1% (w/v) agar, 1% sucrose, and either 1 μM EBR or 0.01% ethanol (solvent for EBR). The plates were kept for 3 days in the dark at 4°C to encourage synchronized germination and then transferred to 22°C with a 16/8 h photoperiod (80 μmol m^-2 ^s^-1^) and allowed to grow for 21 days. For the short-term EBR treatment, 21-day-old seedlings grown in the medium without EBR were submerged for 7 h in sterile water containing 1 μM EBR or 0.01% ethanol.

### Heat stress treatments

The thermotolerance of Arabidopsis seedlings was assayed according to Kagale et al. [12] with minor modifications. Seedlings grown at 22°C for 21 days were exposed to 43°C for 4 h and scored as dead or surviving after 7 days of recovery at 22°C. Seedlings that were completely bleached were considered dead and those with green leaves were considered surviving. Values in Figure 1B represent average percentages of data obtained in three biological replicates. Plant tissue was collected at different time points and quick-frozen for protein isolation and for the TBARS assay.

### Salt stress treatments

Surface sterilized seeds were germinated on 0.5× Murashige and Skoog medium supplemented with B5 vitamins and either 1 μM EBR or 0.01% ethanol. Salt treatment was given by including 150 mM NaCl in the medium. All plates were kept for 3 days in the dark at 4°C plates and then transferred to 22°C. Percent germination was determined 3 days after transferring plates to 22°C. Seeds with emerging cotyledons were scored as germinated. Percent survival was calculated by counting the number of seedlings that showed green leaves at 20 days after imbibition. Values in Figure 5 are average percentages of data obtained in three biological replicates.

### TBARS assay

TBARS assay was performed according to Heath and Packer [34]. Seedlings grown at 22°C for 21 days were subjected to 43°C for 3 h and then allowed to recover for 2 days at 22°C. Seedlings were quick frozen in liquid nitrogen and 0.5 g of the tissue was ground in 1 mL of solution containing buffer 1 and buffer 2 in equal proportions (buffer 1: 0.5 mL of 0.5% (w/v) thibarbituric acid in 20% (v/v) trichloroacetic acid; buffer 2: 0.5 mL 175 mM Nacl in 50 mM Tris, pH 8.0). Ground samples were heated to 94°C for 1 h, centrifuged at 13,000 rpm for 20 min, and the absorbance of the supernatant was measured at 532 and 600 nm. The levels of TBARS were deduced from the malonaldehyde standard curve and in each case the TBARS levels in the EBR-treated samples were compared to that of untreated control. Values in Figure 2A were averaged from three replicates.

### Protein extraction and western blotting

Extraction of total proteins and western blotting were carried out as described by Dhaubhadel et al. [10]. Seedlings grown at 22°C for 21 days were either maintained at 22°C or subjected to 43°C for 3 and 4 h. Seedling tissue above the medium was harvested, frozen in liquid nitrogen and stored at -80°C. Frozen tissue was ground in protein extraction buffer (25 mM Tris-HCL, 1 mM EDTA, 20 mM NaCl, 1 mM PMSF, 1 mM benzamidine, 1 μg/ml leupeptin and 2 μg/ml aprotinin) and after centrifugation at 13,000 rpm for 30 min, the supernatant was transferred to a new tube. Protein concentration was determined by the bradford assay. Total proteins (15 μg) were separated on a 7.5% SDS-polyacrylamide gel and transferred onto nitrocellulose membrane by electroblotting using the Trans-blot Semi-Dry Electrophoretic Transfer Cell (BioRad, Hercules, CA, USA). Hsp90 was detected by sequential incubation with the polyclonal R2 antisera [72] and the peroxidase conjugated anti-rabbit IgG, each at a dilution of 1:5,000, followed by chemiluminescent detection (ECL system, Amersham, Baie d'Urfe, QC). Band densities were quantified by densitometry with NIH ImageJ software http://rsbweb.nih.gov/ij/.

### RT-PCR analysis

RNA was extracted from 21-day-old seedlings grown at 22°C using the SV Total RNA Isolation System (Promega, Madison, WI). Total RNA (3 μg) was reverse transcribed using the oligo (dT)_18 _primer and Super Script First Strand Synthesis System for RT-PCR (Invitrogen, Carlsbad, CA). PCR was carried out with an initial denaturation step of 94°C for 5 min followed by various cycles of denaturation (40 s at 94°C), annealing (45 s at 53°C), and extension (45 s at 72°C). After the last cycle, a final extension was carried out for 5 min at 72°C. PCR was performed for 32 cycles for *PDF1.2, HEL, WRKY70, LTP4, RD22; *35 cycles for *LOX2, PR-1, WAK1, GST1*; and 21 cycles for *ACTIN *(control gene). For quantitative RT-PCR (qRT-PCR) analysis, PCR reactions were performed using SYBR-Green I (Invitrogen, Carlsbad, CA) at 0.1 × concentration and Rotor Gene-3000 thermal cycler (Corbett Research, Sydney, Australia) with an initial denaturation step at 94°C for 4 min followed by various cycles of denaturation (15 s at 94°C), annealing (30 s at 55°C) and extension (30 s at 72°C and 15 s at 83°C). *UBQ10 *was used as control gene for qRT-PCR analysis. The primer sequences used for RT-PCR and qRT-PCR analysis are provided in additional file 3: List of primer sequences used.

## Authors' contributions

**UKD **designed and carried out the experiments and drafted the manuscript. **TR **carried out the experiments involving ABA mutants, participated in manuscript preparation. **PK **is the instigator of the project; she participated in the design of the experiments and contributed to the writing and revision of the paper. All authors have read and approved the final manuscript.

## Supplementary Material

Additional file 1 **Pictures of 21-day-old seedlings**. WT, SA and ABA mutant seedlings grown on a nutrient medium in the absence (C) or presence of 1 μM EBR (E) for 21 days under no-stress conditions. Click here for file

Additional file 2 **Pictures of 3-day-old seedlings**. WT, *ein2*, *eto1-1 *and *npr1-1 *mutant seedlings after 3 days of germination on a nutrient medium in the absence (C) or presence of 1 μM EBR (E) under no-salt conditions. Click here for file

Additional file 3 **List of primer sequences used**. Click here for file
