# Supplementary figures and images for: Brassinosteroid-mediated stress tolerance in Arabidopsis shows interactions with abscisic acid, ethylene and salicylic acid pathways
Source: BMC Plant Biol. 2010 Jul 19;10:151. doi: 10.1186/1471-2229-10-151 (PMC3095295; doi:10.1186/1471-2229-10-151)

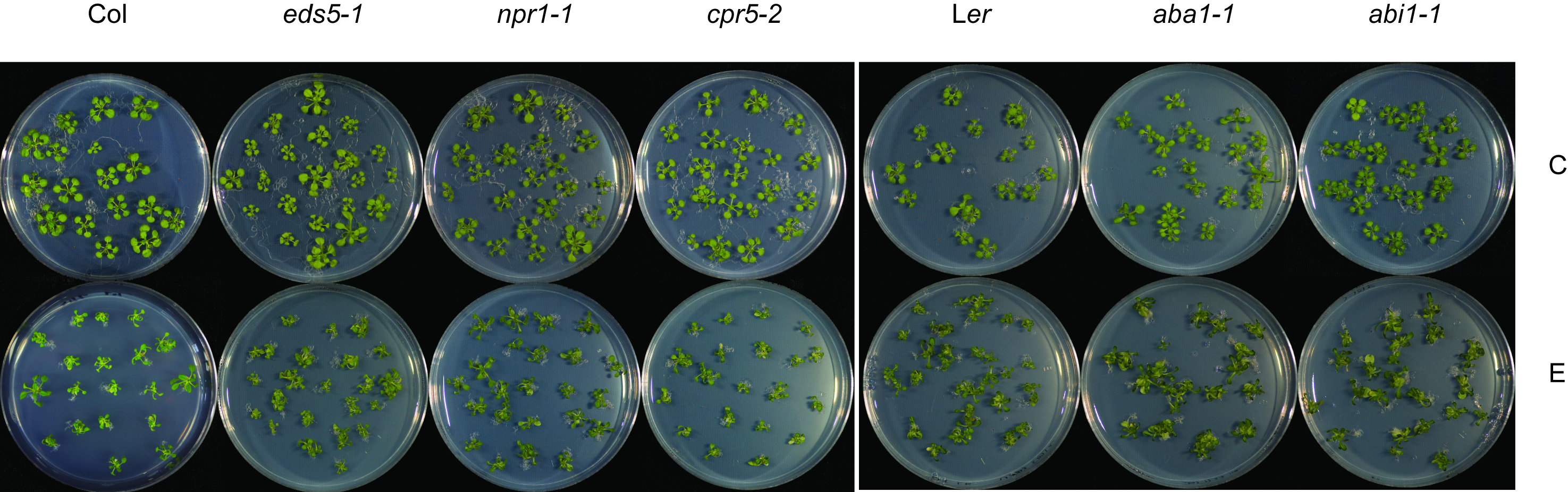

Supplement: Additional file 1 — Pictures of 21-day-old seedlings. WT, SA and ABA mutant seedlings grown on a nutrient medium in the absence (C) or presence of 1 μM EBR (E) for 21 days under no-stress conditions. [file 1471-2229-10-151-S1.JPEG]

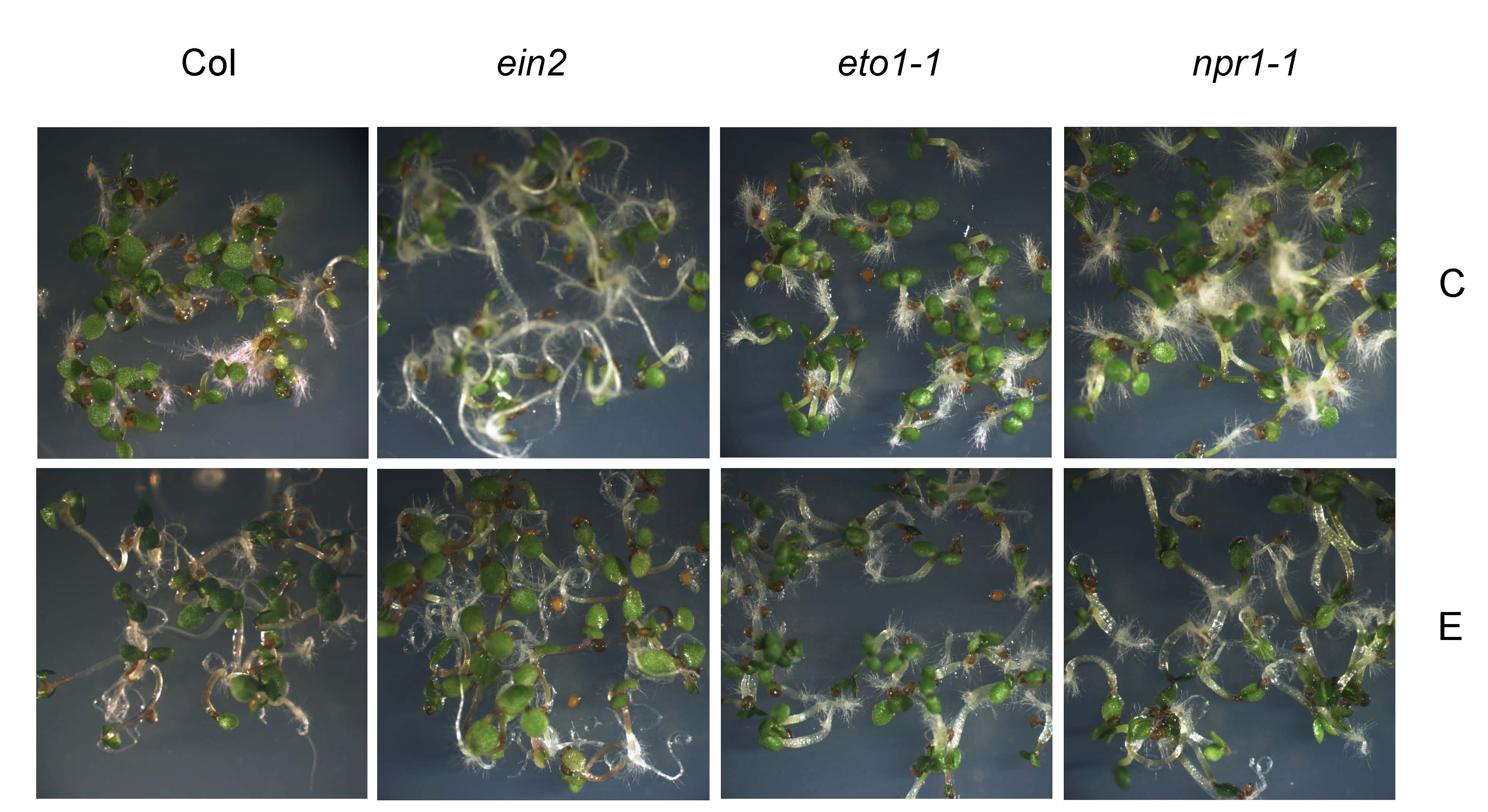

Supplement: Additional file 2 — Pictures of 3-day-old seedlings. WT, ein2, eto1-1 and npr1-1 mutant seedlings after 3 days of germination on a nutrient medium in the absence (C) or presence of 1 μM EBR (E) under no-salt conditions. [file 1471-2229-10-151-S2.JPEG]
