# Supplementary material for: Brassinosteroid-mediated stress tolerance in Arabidopsis shows interactions with abscisic acid, ethylene and salicylic acid pathways
Source: BMC Plant Biol. 2010 Jul 19;10:151. doi: 10.1186/1471-2229-10-151 (PMC3095295; doi:10.1186/1471-2229-10-151)
Supplement: Additional file 3 — List of primer sequences used. [file 1471-2229-10-151-S3.PDF]

Primer sequences used for RT-PCR analysis

| Gene | Forward (5' to 3') | Reverse (5' to 3') |
|---|---|---|
| *PR-1* | GATGTGCCAAAGTGAGGTG | CTGATACATATACACGTCC |
| *WRKY70* | CGCCGCCGTTGAGGGATCTC | CGCCGCCACCTCCAAACAC |
| *WAK1* | GAGTTACTTTGCGACTGCCA | CAGCTTCCTGGATCTCCTTC |
| *PDF1.2* | TCATGGCTAAGTTTGCTTCC | CACACGATTTAGCACCAAAGA |
| *LOX2* | CTCTTCAGAGCACGCTACG | GAAGATGGAGGGAAGAGCTG |
| *HEL* | ACAAGGCCATCTCATTGTTG | GATCAATGGCCGAAACAAG |
| *GST1* | TTGGCTTCTGACCACTTCAC | ACGCTCGTCGAAGAGTTTCT |
| *RD22* | GCGAGCTAAAGCAGTTGCGGTATG | CGGCTAGTAGCTGAACCACACAAC |
| *LTP4* | CACCAACTGCGCCACCATCAAG | GCCATCAAGACAAACAAAGAC |
| *DWF4* | ACGGAGCAAATTCTCGATC | AGCTCTTCAACGGCTTTAG |
| *ACTIN* | TGCTCTTCCTCATGCTAT | ATCCTCCGATCCAGACACTG |

Primer sequences used for qRT-PCR analysis

| Gene | Forward (5' to 3') | Reverse (5' to 3') |
|---|---|---|
| *PR-1* | GATGTGCCAAAGTGAGGTG | TGCATGATCACATCATTACTTC |
| *PDF1.2* | TCATGGCTAAGTTTGCTTCC | TGTCCCACTTGGCTTCTCGC |
| *RD22* | GCGAGCTAAAGCAGTTGCGGTATG | GGGAGGAAGTGGCAGACCGGAAC |
| *GST1* | TTGGCTTCTGACCACTTCAC | ACGCTCGTCGAAGAGTTTCT |
| *DWF4* | ACGGAGCAAATTCTCGATC | AGCTCTTCAACGGCTTTAG |
| *UBQ10* | CAGAACTTTGGCCGACTAC | ATGGTCTTTCCGGTGAGAG |
